# Eco-friendly and sustainable gemini ionic liquid surfactants derived from coffee waste: design, surface activity, oil spill treatment, and antimicrobial performance

**DOI:** 10.1039/d5ra09876a

**Published:** 2026-01-14

**Authors:** Reda Abdel-Hameed, Odeh A. O. Alshammari, Asmaa Hegazy, Eshraqa Ali, Ayham Ali Bani Essa, Maram Ahmed Alhawsawi, Esraa Yasser, Amal M. Metwally, Ghada E. Dawwam, Ahmed H. Tantawy

**Affiliations:** a Basic Science Department, Preparatory Year, University of Ha'il 81442 Hail Saudi Arabia; b Medical and Diagnostic Research Center, University of Ha'il Ha'il 55473 Saudi Arabia; c Department of Chemistry, College of Science, University of Ha'il 81442 Hail Saudi Arabia; d College of Business Administration, Department of Financial Accounting, University of Ha'il 81442 Hail Saudi Arabia; e Chemistry Department, Faculty of Science, Benha University Benha 13518 Egypt aml.metwaly@fsc.bu.edu.eg ahmed.tantawy@fsc.bu.edu.eg; f Botany and Microbiology Department, Faculty of Science, Benha University Benha Egypt

## Abstract

As environmentally safe substitutes for traditional solvents and surfactants, ionic liquids (ILs) have drawn a lot of interest. Among these, IL-based cationic surfactants constitute an important class of multifunctional materials that integrate the unique physicochemical characteristics of ionic liquids with the interfacial activity of surfactants. Due to their dual functionality, they have demonstrated broad applicability as corrosion inhibitors, antimicrobial agents, emulsifiers, and dispersants across the petroleum, pharmaceutical, and environmental sectors. In this case, precursors obtained from natural waste were used to create ionic liquid-based Gemini cationic surfactants (ILGCS) (4a–d). The first of the four primary phases in the synthetic approach was the separation of mixed fatty acids from discarded coffee grounds and culminating in a quaternization reaction to afford the target ILGCS. FT-IR, ^1^H and ^13^C NMR spectroscopy were used to completely describe compounds 4a–d. Their surface tension properties were systematically evaluated, and the ILGCS were further assessed for their efficiency as petro-collecting and petro-dispersing agents, in addition to their antimicrobial activity. The results showed that compound 4d recorded the highest surface activity, characterized by the lowest CMC, the largest surface pressure, and the lowest surface tension at micellization. This result suggests that 4d is a promising candidate for further applications that need strong surface activity, such as emulsification, detergency, or solubilization processes. The petro-collecting and dispersing properties of ILGCS (4a–d) were utilized on three different water sources that differ in salinity. All the synthesized gemini surfactants exhibited outstanding petroleum-dispersing efficiency in fresh and seawater. While in distilled water, the petroleum-collecting properties of the gemini surfactants were observed. Four ionic liquid-based Gemini surfactants (4a–d) were evaluated for antimicrobial activity after 24 h of exposure. Compound 4d (*u* = 6, *m* = 6) showed the highest inhibition zones against *L. monocytogenes* (17 mm) and *S. aureus* (16 mm), indicating strong activity against Gram-positive bacteria. Moderate inhibition was observed for Gram-negative strains *E. coli* (15 mm) and *Salmonella* sp. (14 mm), while *Candida albicans* showed lower susceptibility (13 mm).

## Abstract 2

As environmentally safe substitutes for traditional solvents and surfactants, ionic liquids (ILs) have drawn a lot of interest.

## Graphical abstract

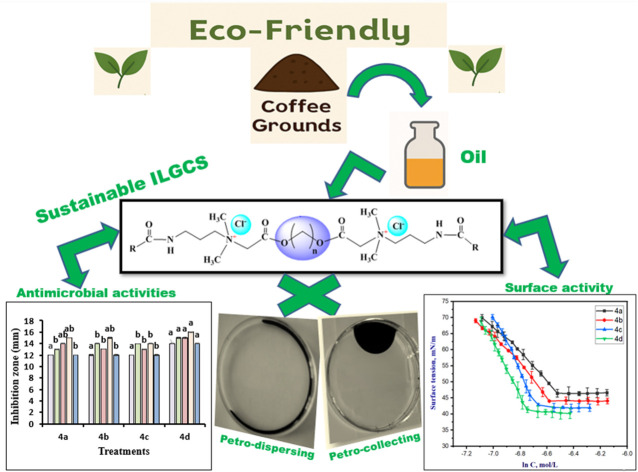

## 1. Introduction

Surface–water pollution caused by accidental petroleum spills represents a serious environmental hazard. Such contamination is characterized by its persistence and the broad spectrum of ecological impacts it imposes. A physical barrier is created when a petroleum layer forms on the water's surface, impeding gaseous exchange and restricting light penetration, thereby adversely affecting aquatic organisms and disrupting overall ecosystem functioning.^1^ Oxygen levels in the water decreased due to the petroleum film preventing the exchange of oxygen between air and water, which negatively affects the life of aquatic organisms.^2^ Surface active materials (surfactants) are one material that plays a vital role in solving this problem, which means petro-collecting and dispersing agents. These materials are characterized by their amphiphilic nature, which can decrease the interfacial tension between petroleum and water that facilitates the formation of fine droplets from petroleum slicks and disperses them throughout the water column.^3^ Also, surfactants can increase the agglomeration of petroleum film and make it easy to remove from the water surface when they work as collecting agents.^4^ Ionic liquids (ILs) and Gemini surfactants are enhancing the emulsification process by reducing the interfacial tension between oil and water.^5^ Gemini surfactants are characterized by the presence of two groups of identical (or non-identical) polar heads (hydrophile) and identical (or different) lengths of alkyl tails (hydrophobic), which allow their characterization into Gemini surfactants with identical hydrophilic heads and hydrophobic tails and those with different heads and tails.^6^ Based on the charge on hydrophilic groups, surfactants are classified into anionic surfactants (*e.g.*, alkyl sulfates, sulfonates, carboxylates), which dominate the detergent industry; cationic surfactants (*e.g.*, quaternary ammonium compounds), that have attracted in their uses in antimicrobial and conditioning applications; nonionic surfactants (*e.g.*, alcohol ethoxylates, sorbitan esters), valued for their mildness and stability; and zwitterionic surfactants (*e.g.*, betaines), which have both positive and negative charges in the same molecule and display unique compatibility profiles.^7,8^

Recently, all studies focused on the development of ILs and surfactants as eco-friendly systems for removing petroleum films from water surfaces.^9–13^

ILs as environmentally friendly alternatives have attracted greater attention than traditional solvents and surfactants due to their characteristic physicochemical properties, including thermal stability, adjustable polarity, and negligible vapor pressure.^14,15^ Through the introduction of ILs fragments into surfactant molecules developed by a new class that is known as ILs-based cationic surfactants. ILs-based cationic surfactants are a unique type of multifunctional substances that combine between characteristic properties of surfactants and the unique properties of ILs.^16^ They exhibited observable surface activity and low critical micelle concentrations (CMC) in addition to the enhancement in solubilization in comparison with traditional surfactants of quaternary ammonium moiety.^17^ The flexibility in the structure of ILs-based cationic surfactants allows for introducing classes of cations like ammonium, phosphonium, pyridinium, and imidazolium for adjusting the hydrophilic–lipophilic balance.^18,19^ In recent studies, a wide range of their applications are utilized as antimicrobial agents, corrosion inhibitors, dispersants in petroleum, and emulsifiers in addition to their applications in fields of pharmaceutical and environmental.^20,21^

Recently, IL-based Gemini cationic surfactants have been created because of their better interfacial characteristics, improved solubilization, and application performance.^22–24^ Gemini surfactants are a unique type of amphiphilic substances containing two hydrophilic groups (head) and two hydrophobic groups (tails) that are connected through a spacer. Gemini surfactants differ from monomeric surfactants, which have low CMC, foaming properties, high solubility and higher surface activity that enhance their efficiency in different applications.^25,26^ A potential range of their applications was demonstrated in their antimicrobial activity,^27^ drug and gene delivery systems,^28^ enhanced oil recovery,^29^ and corrosion inhibition.^30^ Gemini surfactants become highlighted by researchers because their physicochemical properties can be adjusted by the nature of hydrophilic groups, spacer units, and length of hydrophobic chain.^31^ Cationic Gemini surfactants with a head group having positive charges, such as quaternary ammonium ions, attracted attention due to antimicrobial efficiency, which interacted with negatively charged surfaces and biological membranes.^32^ Salts of quaternary ammonium, imidazolium, and pyridinium are toxic to pathogenic bacteria and fungi.^33^ Their toxicity is enhanced by increasing the length of the alkyl chain and spacer between two head groups.^34,35^

Developing bio-based products with materials derived from renewable and natural resources, such as biomass, is a more environmentally friendly strategy that has the potential to significantly reduce CO_2_ emissions.^36,37^ In daily life, surfactants are among the most prevalent compounds produced from petrochemicals. Because of their ability to reduce surface or interfacial tension between two liquids, these chemicals are widely utilized in the food, pharmaceutical, cosmetics, and detergent sectors.^38–40^

This study presents the synthesis and evaluation of four ionic liquid-based Gemini cationic surfactants (ILGCS) (4a–d) for their performance as petro-collecting and petro-dispersing agents. In addition, the antimicrobial activity of the ILGCS was assessed. The structures of compounds 4a–d were confirmed using FT-IR, as well as ^1^H, and ^13^C NMR spectroscopy. Their surface-active properties were also systematically investigated. The petro-collecting and dispersing properties of ILGCS (4a–d) were utilized on three different water sources that differ in salinity. The antimicrobial activity of four ionic liquid-based Gemini surfactants (4a–d) was assessed against Gram-positive strains (*Listeria monocytogenes* ATCC 19155 and *Staphylococcus aureus* ATCC 43300), Gram-negative strains (*Escherichia coli* ATCC 8739 and *Salmonella* sp. ATCC 14028), and the unicellular fungus *Candida albicans* ATCC 10231.

## 2. Materials and synthesis

### 2.1. Materials

Spent coffee grounds were collected as waste products from coffee. From Sigma-Aldrich, ethane-1,2-diol and monochloroacetic acid were obtained. Diols derivatives (propane-1,3-diol, pentane-1,5-diol, and hexane-1,6-diol), triethyl amine (TEA), *N*,*N*-dimethyl propane-1,3-diamine, thionyl chloride, and *p*-toluene sulphonic acid (*p*-TsOH) were supplied by Fisher Scientific company. From El-Nasr Pharmaceutical Chemical Co, tetrahydrofuran (THF), dichloromethane (DCM), dimethylformamide (DMF), benzene, methanol, and sulphuric acid were obtained. A Thermo-Scientific Nicolet iS10 FT-IR spectrometer is a technique to provide FT-IR spectra of all obtained materials. Bruker Avance 400 MHz instrument also provides both of ^1^H and ^13^C NMR spectra. PROFILENC TECHNOLOGIES ECS 8020 CHNS-O elemental combustion system – elemental analyzer was used through giving the percentage of elements that found in final products.

### 2.2. Synthesis

#### 2.2.1. Isolation of mixed fatty acids (MFAs) from waste of spent coffee

Based on ref. 41, MFAs was obtained. Soxhlet extractor was used to extract the oil from the wastes of spent coffee, which wastes spent coffee in *n*-hexane undergoing reflux for 7 h. The isolated oils were obtained by rotatory evaporation of *n*-hexane. The obtained oil undergoes saponification with a solution of alcoholic potassium hydroxide and then is acidified with sulphuric acid to obtain MFAs.

#### 2.2.2. Synthesis of acid chloride of mixed fatty acids (ACMFAs) (1)

10 mmol of thionyl chloride in 5 mL of DCM and a few drops of DMF as a catalyst were added to 1.01 mmol of MFAs, which the reaction undergoes stirring at room temperature for 4 h. ACMFAs (1) was obtained *via* evaporating the excess of thionyl chloride and DCM after the completion of reaction. Compound 1 is obtained as a pale brown viscous material with a yield of 85%.

#### 2.2.3. Synthesis of *N*-(3-(dimethylamino)propylalkamide)

Compound 1 was dissolved in THF, and a few drops of TEA were added as a catalyst. The reaction was kept for stirring under cooling, and then *N*,*N*-dimethyl propane-1,3-diamine was added. After completing the reaction, the white precipitate of *N*,*N*-dicyclohexyl urea was removed. *N*-(3-(Dimethylamino)propyl)alkamide was obtained by extraction of the filtrate with saturated solution of sodium carbonate and DCM. The solvent was evaporated, leaving pale yellow solid material of *N*-(3-(dimethylamino)propyl)alkamide (2) with a yield of 88%.

**Fig. 1:**
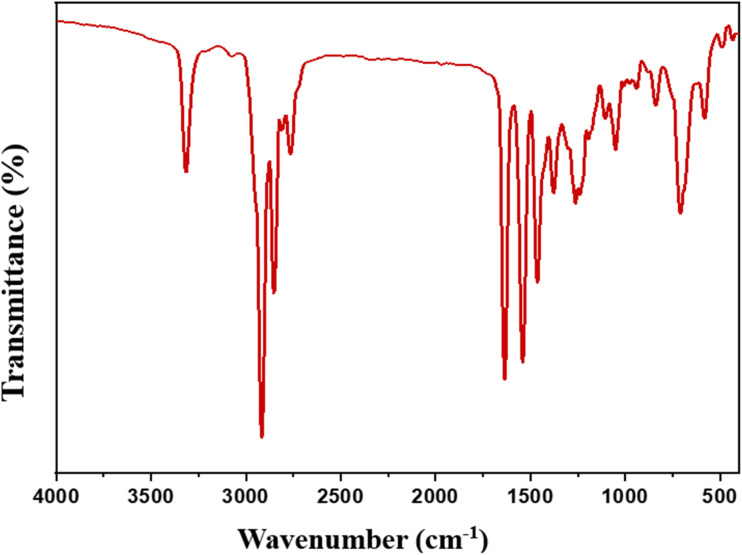
FT-IR spectrum for *N*-(3-(dimethylamino)propyl)alkamide (2).

Spectrum of FT-IR (*ν*/cm^−1^) (Fig. 1) for compound (2) at: 3315 cm^−1^ (N–H bond), 2852, 2916 cm^−1^ (asymmetric and symmetric C–H), 1635 cm^−1^ (C=O of –NH–C=O), 1246 cm^−1^ (C–N bond).

#### 2.2.4. Synthesis of derivatives of alkane bis(2-chloroacetate)

Compounds (3a–d) were obtained according to ref. 30, *p*-TsOH as a catalyst and a mixture of monochloroacetic acid with ethane-1,2-diol that dissolved in dry benzene were refluxed under Dean Stark apparatus. By using saturation solution of sodium carbonates, ethane-1,2-diyl bis(2-chloroacetate) 3a was isolated. After the evaporation of benzene, Compound 3a was obtained. Compounds 3b, 3c, and 3d were obtained with the same procedure but differ in using diols compounds.

**Fig. 2:**
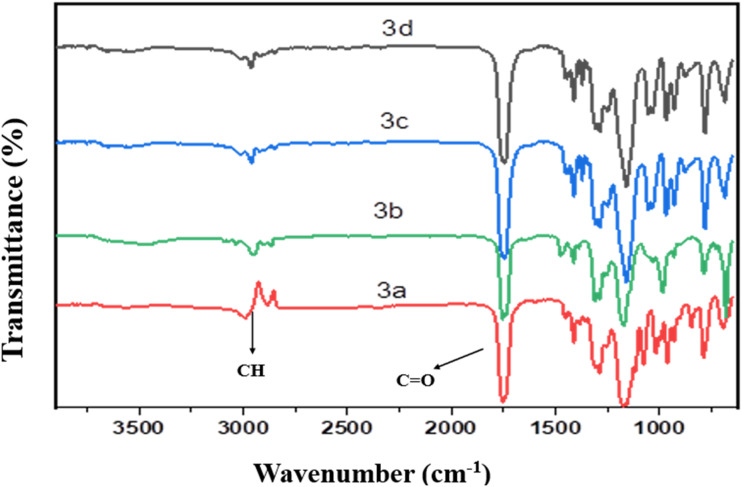
FT-IR spectra for derivatives of alkane bis(2-chloroacetate) (3a–d).

Spectra of FT-IR (*ν*/cm^−1^) for compounds (3a–d) (Fig. 2a–d) at: 2858–2860, 2928–2960 cm^−1^ (asymmetric and symmetric C–H), 1746–1750 cm^−1^ (C=O of ester), 1158–1197 cm^−1^ (C–O), 781–788 cm^−1^ (C–Cl).

#### 2.2.5. Synthesis of ILGCS

ILGCS (4a–d) were obtained by heating a mixture of compound 2 with separate compounds 3a–d in sealed tubes with adding methanol for 96 h. The product was obtained after evaporation the methanol and several times was purified *via* using diethyl ether. ILGCS (4a–d) were obtained with ranging in color from brown to pale yellow.

**Fig. 3:**
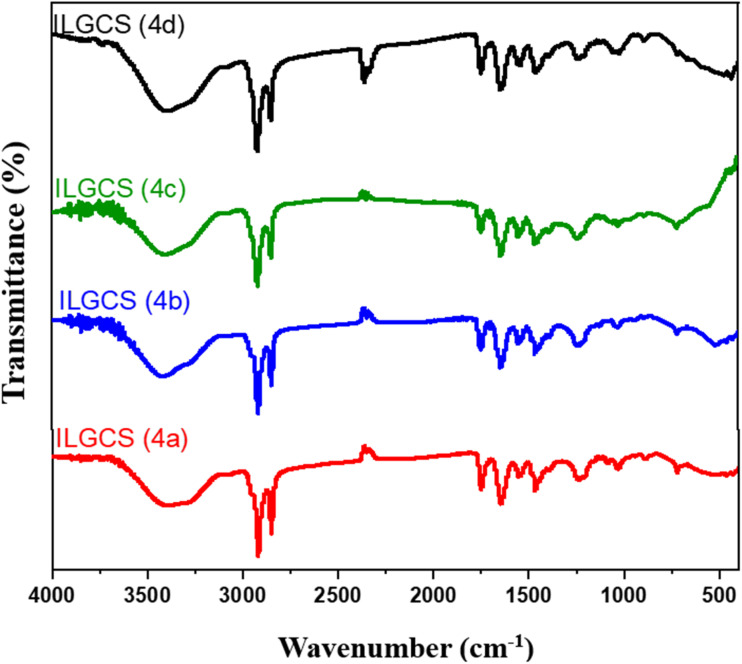
FT-IR spectra for ILGCS (4a–d).

Spectra of FT-IR (*ν*/cm^−1^) for ILGCS (4a–d) (Fig. 3a–d) at: 2849–2852, 2917–2922 cm^−1^ (asymmetric and symmetric C–H), 1748–1749 cm^−1^ (C=O of ester), 1646–1647 cm^−1^ (C=O of –NH–C=O), 1235–1246 cm^−1^ (C–N bond), and 1020–1230 cm^−1^ (C–O bond).

##### 2.2.5.1. *N*,*N*′-((Ethane-1,2-diylbis(oxy))bis(2-oxoethane-2,1-diyl))bis(*N*,*N*-dimethyl-3-tetradecanamidopropan-1-aminium)chloride (4a)

^1^H NMR (500 MHz, DMSO-*d*_6_) *δ* 8.12 (s, 2H), 4.50 (s, 4H), 3.70 (s, 4H), 3.51–3.47 (m, 4H), 3.18 (s, 12H), 3.03 (dd, *J* = 11.9, 6.1 Hz, 4H), 2.01 (t, *J* = 7.5 Hz, 4H), 1.82–1.71 (m, 4H), 1.42 (d, *J* = 6.5 Hz, 4H), 1.17 (s, 54H), 0.79 (t, *J* = 6.8 Hz, 6H). ^13^C NMR (126 MHz, DMSO-*d*_6_) *δ* 172.95, 165.82, 63.34, 63.00, 60.98, 53.25, 51.55, 35.91, 35.81, 31.83, 29.60, 29.55, 29.50, 29.41, 29.30, 29.25, 25.74, 23.13, 22.62, 14.44 (Fig. S1).

##### 2.2.5.2. *N*,*N*′-((Propane-1,3-diylbis(oxy))bis(2-oxoethane-2,1-diyl))bis(*N*,*N*-dimethyl-3-tetradecanamidopropan-1-aminium)chloride (4b)

^1^H NMR (500 MHz, DMSO-*d*_6_) *δ* 8.12 (d, *J* = 5.5 Hz, 2H), 4.49 (s, 4H), 3.70 (s, 5H), 3.49 (s, 4H), 3.17 (s, 12H), 3.02 (d, *J* = 6.1 Hz, 4H), 2.01 (t, *J* = 7.5 Hz, 4H), 1.77 (dd, *J* = 15.2, 7.2 Hz, 4H), 1.42 (d, *J* = 6.5 Hz, 4H), 1.18 (s, 54H), 0.79 (t, *J* = 6.8 Hz, 6H). ^13^C NMR (126 MHz, DMSO-*d*_6_) *δ* 173.83, 165.23, 64.02, 62.16, 60.23, 52.46, 50.26, 41.11, 38.70, 34.91, 31.19, 30.09, 26.03, 23.35, 22.58, 14.20 (Fig. S2).

##### 2.2.5.3. *N*,*N′*-((Pentane-1,5-diylbis(oxy))bis(2-oxoethane-2,1-diyl))bis(*N*,*N*-dimethyl-3-tetradecanamidopropan-1-aminium)chloride (4c)

^1^H NMR (500 MHz, DMSO-*d*_6_) *δ* 8.06 (d, *J* = 25.1 Hz, 2H), 4.47 (s, 2H), 3.82 (s, 2H), 3.70 (s, 6H), 3.17 (s, 7H), 3.09 (s, 4H), 3.03 (d, *J* = 6.1 Hz, 4H), 2.65 (d, *J* = 9.9 Hz, 2H), 2.05–1.97 (m, 4H), 1.76 (ddd, *J* = 22.5, 12.2, 6.9 Hz, 4H), 1.42 (d, *J* = 6.4 Hz, 4H), 1.19 (d, *J* = 15.0 Hz, 52H), 0.79 (t, *J* = 6.9 Hz, 6H). ^13^C NMR (126 MHz, DMSO-*d*_6_) *δ* 172.96, 165.81, 63.01, 61.25, 60.99, 53.27, 51.58, 50.76, 42.37, 35.92, 35.81, 32.95, 31.83, 29.60, 29.55, 29.51, 29.50, 29.40, 29.37, 29.30, 29.28, 29.25, 25.76, 25.74, 24.69, 23.20, 23.14, 22.62, 22.54, 14.46 (Fig. S3).

##### 2.2.5.4 *N*,*N*′-((Hexane-1,6-diylbis(oxy))bis(2-oxoethane-2,1-diyl))bis(*N*,*N*-dimethyl-3-tetradecanamidopropan-1-aminium)chloride (4d)

^1^H NMR (500 MHz, DMSO-*d*_6_) *δ* 8.06 (d, *J* = 25.1 Hz, 2H), 4.47 (s, 2H), 3.70 (s, 4H), 3.53 (d, *J* = 19.9 Hz, 5H), 3.13 (d, *J* = 36.8 Hz, 12H), 3.04–2.96 (m, 4H), 2.06–1.97 (m, 4H), 1.76 (ddd, *J* = 22.5, 12.2, 6.9 Hz, 4H), 1.42 (d, *J* = 6.4 Hz, 4H), 1.18 (s, 51H), 0.79 (t, *J* = 6.9 Hz, 6H). ^13^C NMR (126 MHz, DMSO-*d*_6_) *δ* 173.05, 165.93, 63.46, 60.76, 55.20, 53.77, 51.81, 50.62, 42.66, 36.33, 35.46, 33.40, 32.09, 30.03, 28.81, 25.88, 24.35, 23.26, 22.93, 14.31 (Fig. S4).

### 2.3. Surface properties

At 25 °C (room temperature), the surface tension values of freshly prepared solutions of compounds 4a–d were determined using a Krüss K6 tensiometer. The solutions were allowed to stand for 24 hours at room temperature to ensure equilibrium before measurement. Before each determination, the platinum ring was thoroughly rinsed several times with distilled water to eliminate any residual surface impurities. For each sample, three consecutive measurements were taken to ensure repeatability, and the average value was recorded.^42^

### 2.4. Petroleum studies

It was discovered that the new class of chemicals was soluble in water. We examined their capacity to disseminate and gather petroleum films in two forms (solid and 2.5% aqueous solution) over the surface of water with three types of water differ in salinity. The Red Sea of South Sinai, Egypt provided the crude petroleum oil with a density of 0.85 g cm^−3^ and a viscosity of 0.17 cm^2^ s^1^ at 293 K. A 2.5% aqueous solution or 0.01 g of each created compound were applied to thin layer of crude oil of 0.14 mm thick on the three water's surfaces differ in salinity. *Via* using the formula *K* = *S*_o_/*S*, *K* was calculated which represents the petro-collecting behavior. *S*_o_ and *S* are the surface areas of the petroleum film at the start of the experiment and produced under the effect of the synthesized compounds, respectively. Through the monitoring process, the film’ surface area was regularly measured, and *K* values were calculated at predefined intervals (*τ*). *K*_D_ represents petroleum-dispersing ability, which can calculate *via* the ratio of the surface area of clean water to the initial area of petroleum film.^9,11^

### 2.5. Antimicrobial activity

The antimicrobial activity of the synthesized ILGCS (4a–d) was evaluated against Gram-positive strains (*Staphylococcus aureus* ATCC 43300 and *Listeria monocytogenes* ATCC 19155), Gram-negative strains (*Escherichia coli* ATCC 8739 and *Salmonella* sp. ATCC 14028), and the unicellular fungus *Candida albicans* ATCC 10231 using the agar well diffusion method, as described by Dawwam *et al.*^43^ Bacterial cultures were grown in nutrient broth under agitation at 200 rpm for 24 h at 37 °C. Following incubation, 1.5 × 10^8^ CFU of each strain were inoculated onto Mueller–Hinton agar plates. Wells of 7 mm diameter were prepared in the agar, into which 200 µL of each test compound was introduced. Every experiment was carried out in triplicate, and plates were incubated at 37 °C for 24 h. The inhibitory zone diameters (mm) were used to measure the antimicrobial activity.

### 2.6. Statistical analysis

Statistical analyses were conducted using IBM® SPSS® Statistics software (version 21). Duncan's multiple range test was applied at a 5% significance level, and all experiments were performed in triplicate.

## 3. Result and discussion

### 3.1. Synthesis

**Scheme 1:**
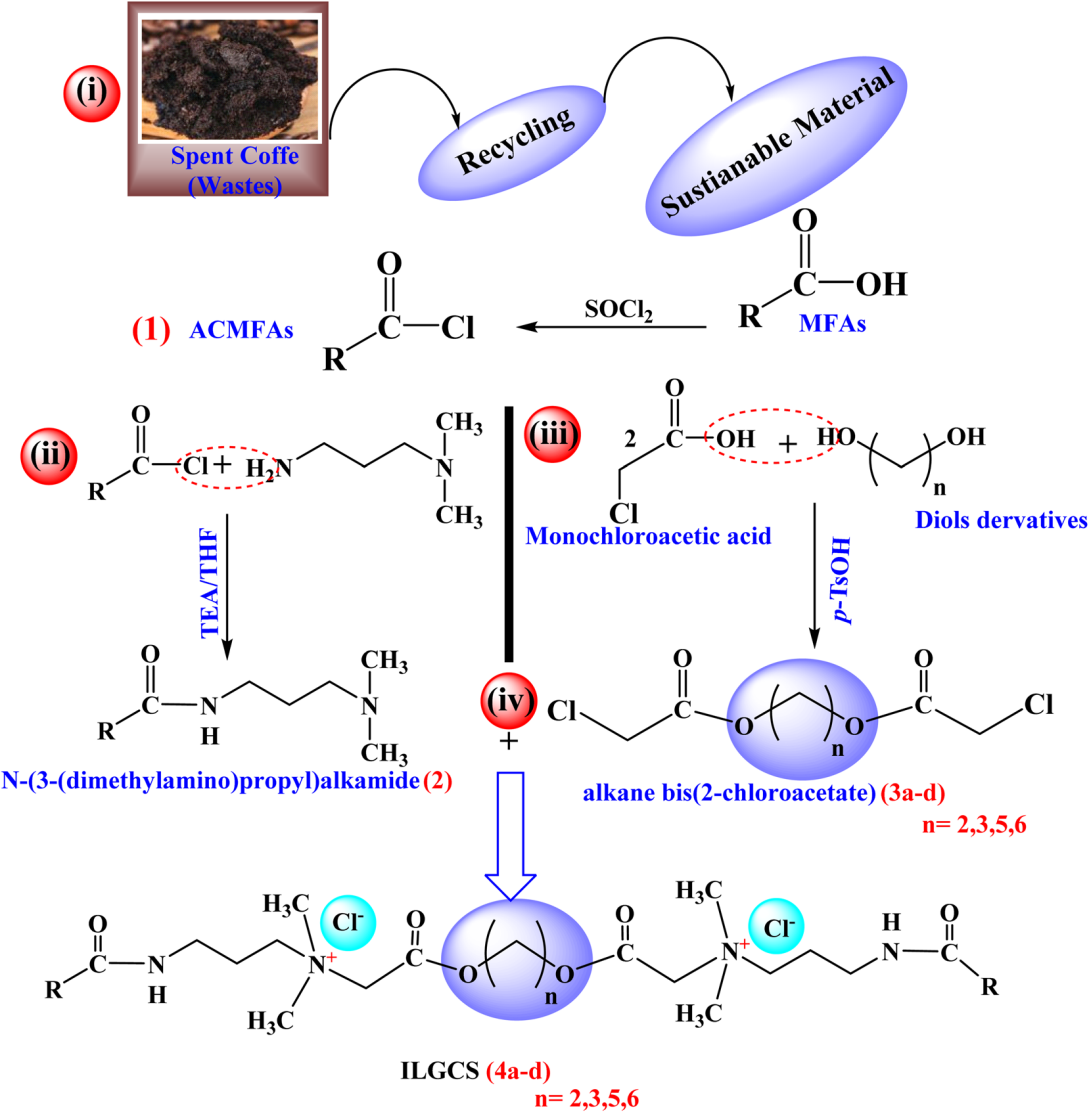
Systematic route for synthesis of ILGCS (4a–d).

By recycling spent coffee waste, a new series of ionic liquid–based Gemini cationic surfactants (ILGCS) (4a–d) was successfully developed. These surfactants exhibited remarkable antimicrobial efficiency, along with excellent performance as oil-spill collecting and dispersing agents. The synthetic pathway employed for their preparation was systematically designed and is illustrated in Scheme 1. The way of the synthesis passes through four main steps. Firstly, acid chloride of mixed fatty acids (ACMFAs) (1) was obtained by treating mixed fatty acids isolated from wastes of spent coffee with thionyl chloride. The second step includes the treatment of compound (1) with *N*,*N*-dimethyl propane-1,3-diamine to obtain *N*-(3-(dimethylamino)propyl)alkamide (2), while in third step, derivatives of diols compounds reacted with monochloroacetic acid to obtain derivatives of alkane bis(2-chloroacetate) (3a–d). Finally, ILGCS (4a–d) were obtained by reaction of compound (2) with derivatives of alkane bis(2-chloroacetate) (3a–d).

Through the spectroscopic tools such as FT-IR, ^1^H, and ^13^C NMR, the structure of the produced materials was confirmed. The reaction was followed by a study of FT-IR for each compound produced in each step. Compound 2 showed an important band at 1635 cm^−1^ in FT-IR spectrum (Fig. 1) due to the formation of C=O of the formed amide in the step, which acid chloride of mixed fatty acids (ACMFAs) (1)was reacted with *N*,*N*-dimethyl propane-1,3-diamine. Compounds 3a–d showed characteristic bands in the range of 1746–1750 cm^−1^ for carbonyl of the formed ester in FT-IR spectra (Fig. 2a–d), which recognized the reaction between monochloroacetic acid with diols derivatives. The final products for ILGCS (4a–d) were reported *via* FT-IR and ^1^H NMR spectra. In Fig. 3(a and b), both bands for carbonyl groups of ester and amide appeared at 1749 cm^−1^ and 1647 cm^−1^, respectively. This recognized that *N*-(3-(dimethylamino)propyl)alkamide (2) was quanternized *via* alkane bis(2-chloroacetate) (3a–d).

Table 1 summarizes the elemental analysis data for the synthesized Gemini surfactants (4a–d). The predicted and experimental values for carbon (C), hydrogen (H), nitrogen (N), chlorine (Cl), and oxygen (O) are in good agreement. The effective synthesis and high purity of the obtained compounds are clearly supported by this close relationship.

**Table 1:** Elemental analysis of the synthesized Gemini surfactant (4a–d)

Compounds	C		H		N		Cl		O	
	Calc.	Exp.	Calc.	Exp.	Calc.	Exp.	Calc.	Exp.	Calc.	Exp.
4a	64.98	63.9 ± 0.3	10.91	10.1 ± 0.2	6.06	5.9 ± 0.2	7.67	7.4 ± 0.5	10.39	10.1
4b	65.28	64.6 ± 0.3	10.96	10.2 ± 0.2	5.97	5.7 ± 0.2	7.56	7.2 ± 0.5	10.23	9.8
4c	65.87	65.1 ± 0.3	11.06	10.5 ± 0.2	5.80	5.3 ± 0.2	7.34	6.9 ± 0.5	9.93	9.2

### 3.2. Surface properties

The surface activity of the synthesized surfactants (4a–d) was evaluated using their critical micelle concentration (CMC), the surface tension at CMC (*γ*_CMC_), and the surface pressure at CMC (effectiveness, *Π*_CMC_). These results demonstrate a steady and methodical pattern throughout the series, demonstrating that the structural alterations made to the compounds successfully improve their surface-active characteristics.

**Fig. 4:**
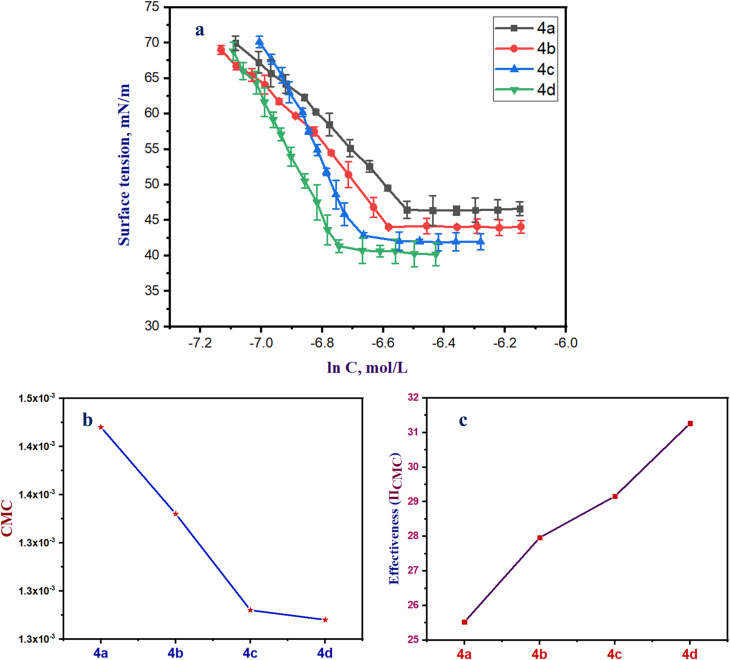
(a) Surface tension of the synthesized Gemini surfactants (4a–d, mN m^−1^) *versus* concentration (mol L^−1^), (b) the CMC values against the synthesized Gemini surfactants (4a–d, mol L^−1^), and (c) the effectiveness of the synthesized Gemini surfactants (4a–d, mN m^−1^).

Exhibits a continual drop in surface tension with increasing concentration until reaching a plateau at the critical micelle concentration (CMC), which is characteristic for amphiphilic surfactants (Fig. 4a).

As shown in Fig. 4a, the observed difference in surface tension between compounds 4a, 4b, and 4c can be attributed to variations in their molecular structures and aggregation behavior at different concentrations. At low concentrations, compound 4c exhibits stronger intermolecular interactions at the air–water interface, leading to higher surface tension compared to 4a and 4b. However, as the concentration increases, 4c tends to form micelles or aggregates more readily, thereby reducing the number of free molecules available at the interface. This results in a faster decrease in surface tension relative to 4a and 4b at higher concentrations.

Furthermore, the sharper surface tension drop of 4c near the critical micelle concentration (CMC) indicates a higher surface excess concentration and better interfacial organization at elevated concentrations. This dual behavior—lower efficiency at very low concentration but superior performance at higher concentration—is commonly observed for surfactants with stronger hydrophobic character or larger molecular size, where adsorption becomes more favorable only after a threshold concentration is reached.

Interestingly, there is a noticeable drop in CMC from 4a to 4d (4a > 4b > 4c > 4d), as seen in Fig. 4b. Compound 4d has the greatest capacity to self-assemble in aqueous solution, as evidenced by its lowest CMC (0.00127 M). As the series proceeds on, increasing hydrophobicity or better molecular packing is reflected in the declining CMC values. The above trend demonstrates that the structural evolution from 4a to 4d increases the thermodynamic driving force for micellization, most likely as a result of the hydrophobic moiety's better interaction with the aqueous environment.

It is evident from the measured *γ*_CMC_ values how well each surfactant lowers the water's surface free energy. Surfactant molecules are mostly adsorbed at the air–water interface at low concentrations. The surface tension gradually decreases as concentration rises because more molecules form up at the interface. This continues until the surface becomes saturated with surfactant molecules. Once the CMC is reached, additional surfactant molecules no longer adsorb at the interface but instead form micelles in the bulk solution. As consequently, surface tension becomes almost constant at *γ*_CMC_, which represents the lowest surface tension that each surfactant can achieve.

Fig. 4c exhibited that the *γ*_CMC_ values also decrease systematically from 4a (46.47 mN m^−1^) to 4d (40.74 mN m^−1^). This reduction in surface tension at the point of micellization indicates that compounds with lower CMC values are more efficient at reducing the surface free energy of water. The stronger surface tension-lowering ability of 4d highlights its superior efficiency in orienting at the air–water interface and disrupting water–water hydrogen bonding, compared to the earlier members of the series. This behavior is typical of surfactants with stronger hydrophobic interactions and more favorable interfacial packing. The surface pressure at CMC (*Π*_CMC_ = *γ*_0_ − *γ*_CMC_) raises progressively from 4a to 4d, with values ranging from 25.53 to 31.27 mN m^−1^. The increasing *Π*_CMC_ values confirm that later members of the series exert a stronger adsorption effect at the interface. Higher *Π*_CMC_ values indicate more effective displacement of water molecules at the surface and greater molecular packing. This again supports the conclusion that compound 4d possesses the highest surface activity.

With the lowest CMC, the highest surface pressure, and the lowest surface tension at micellization, compound 4d exhibits the highest surface activity of the four compounds. These findings demonstrate how effectively structural design enhances surfactant performance and indicate that 4d is the most viable option for additional applications that call for high surface activity, including emulsification, detergency, or solubilization processes.

### 3.3. Petroleum studies (petro-dispersing and petro-collecting abilities of the synthesized surfactant)

**Fig. 5:**
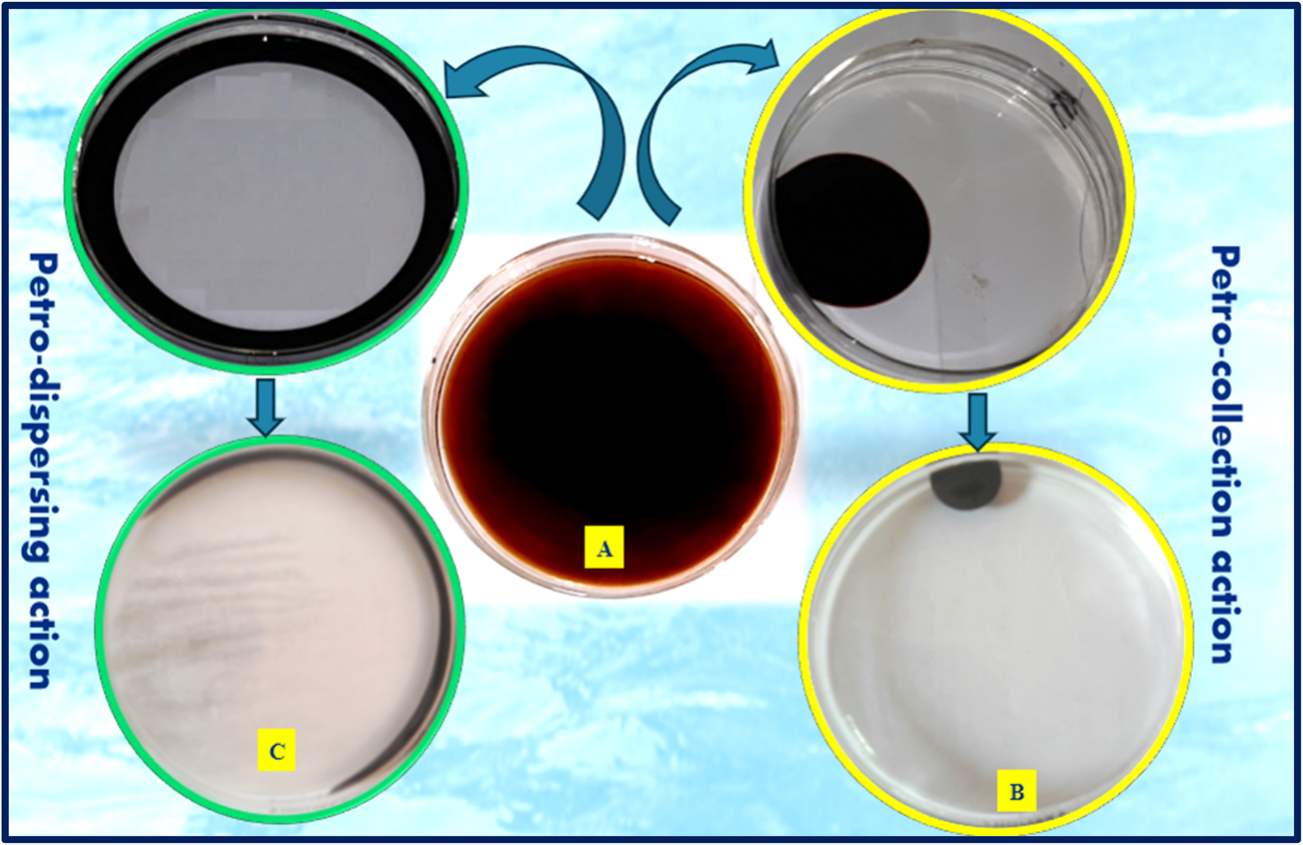
Effect of the synthesized ionic liquid (4d) on petroleum (A) untreated thin film, (B) petro-collecting, and (C) petro-dispersing.

Accidents involving oil tankers, pipelines, and petroleum transportation often lead to the contamination of water surfaces with oil.^1^ Although mechanical methods can remove thick oil slicks, thin oil films inevitably remain on the water surface and cannot be eliminated through such techniques. These films pose serious environmental hazards, as they hinder oxygen exchange and block sunlight penetration into deeper water layers, thereby disrupting marine ecosystems and endangering aquatic life.^2^ Consequently, the use of oil-collecting or dispersing agents is regarded as the most effective and practical approach for removing these residual thin oil films. The petro-dispersing and petro-collecting efficiencies of the synthesized gemini surfactants are presented in Table 2 and Fig. 5. These evaluations were performed in 2.5 wt% aqueous solutions. The gemini surfactants were shown to be more effective as petro-dispersing agents than as petro-collecting agents in the diluted state, with the length of the spacer group having an impact on their performance. Interestingly, all the as-prepared gemini surfactants showed outstanding petroleum-dispersing efficiency in freshwater. The dispersion coefficient (*K*_D_) ranged from 94.5 to 93.3% for compound 4a and from 97.8 to 95.5% for compound 4d, while compounds 4b and 4c maintained stable and consistent dispersing action for more than three days. In seawater, all the as-prepared gemini surfactants demonstrated petroleum-dispersing activity, with compound 4c exhibiting superior performance compared to the other surfactants, which showed variations in efficiency over time. The petroleum-collecting properties of the gemini surfactants were noted in distilled water, with *K* values ranging from 9.87 to 8.60 and 5.36. However, these collecting effects were limited to the first 24 hours, after which the petro-dispersing properties became dominant and persisted for more than three days.

**Table 2:** Petro-collecting/dispersing properties of the synthesized Gemini surfactant (4a–d)

Compd. no.	Undiluted product						2.5% wt water solution					
	Distilled water		Fresh water		Sea water		Distilled water		Fresh water		Sea water	
	*τ* (h)	*K* (*k*_D_)	*τ* (h)	*K* (*k*_D_)	*τ* (h)	*K* (*k*_D_)	*τ* (h)	*K* (*k*_D_)	*τ* (h)	*K* (*k*_D_)	*τ* (h)	*K* (*k*_D_)
4a	0–12	6.77	0–12	94.5%	0–12	95.5%	0–12	5.36	0–12	94.5%	0–12	95.5%
	24	NC^a^	24	NC^a^	24	NC^a^	24	NC^a^	24	NC^a^	24	NC^a^
	48–72	91.1%	48–72	95.5%	48–72	NC^a^	48–72	84.7%	48–72	93.3%	48–72	93.3%
	72–96	NC^a^	72–96	NC^a^	72–96	NC^a^	72–96	NC^a^	72–96	NC^a^	72–96	NC^a^
4b	0–12	8.45	0–12	97.8%	0–12	93.2%	0–12	9.87	0–12	95.6%	0–12	91.1%
	24	NC^a^	24	NC^a^	24	NC^a^	24	NC^a^	24	NC^a^	24	NC^a^
	48–72	95.5%	48–72	NC^a^	48–72	85.7%	48–72	95.5%	48–72	NC^a^	48–72	78.6%
	72–96	NC^a^	72–96	NC^a^	72–96	NC^a^	72–96	NC^a^	72–96	NC^a^	72–96	NC^a^
4c	0–12	10.67	0–12	97.8%	0–12	97.8%	0–12	9.87	0–12	97.8%	0–12	95.5%
	24	NC^a^	24	NC^a^	24	NC^a^	24	NC^a^	24	NC^a^	24	NC^a^
	48–72	96.3%	48–72	NC^a^	48–72	NC^a^	48–72	94.4%	48–72	NC^a^	48–72	NC^a^
	72–96	NC^a^	72–96	NC^a^	72–96	NC^a^	72–96	NC^a^	72–96	NC^a^	72–96	NC^a^
4d	0–12	9.11	0–12	95.4%	0–12	93.5%	0–12	8.60	0–12	96.8%	0–12	95.5%
	24	95.2%	24	NC^a^	24	NC^a^	24	NC^a^	24	NC^a^	24	NC^a^
	48–72	NC^a^	48–72	97.4%	48–72	97.7%	48–72	98.1%	48–72	97.5%	48–72	97.7%
	72–96	NC^a^	72–96	NC^a^	72–96	NC^a^	72–96	NC^a^	72–96	NC^a^	72–96	NC^a^

a NC = no change.

Interestingly, based on the results of petroleum indices, the effect of spacer length was found. Whereas the Short spacers (*e.g.*, 4a) exhibited moderate dispersing ability and relatively low stability over time. Also, the medium-length spacers (4b and 4c) showed the best balance between hydrophilic and hydrophobic interactions, enabling both strong surface adsorption and effective micelle formation. Then, the longer spacer (4d) maintained high *K*_D_ values, particularly in seawater, but the effect plateaued, suggesting a limit beyond which further elongation of the spacer does not enhance performance.

As well as the time-dependent behavior study showed that the temporal evolution of petro-dispersing and collecting properties shows two distinct phases. Initially, at 0–12 h, the high collecting activity was revealed due to a rapid adsorption and aggregation at the oil–water interface. Later, at 24–96 h, the dominance of dispersion was obtained due to a micelle stabilization and reduction in interfacial tension, leading to the formation of a stable oil-in-water emulsion. The persistence of dispersing efficiency for up to 72 hours indicates that the synthesized gemini surfactants form thermodynamically stable systems, a crucial factor for practical applications in oil spill remediation.

The synthesized gemini surfactants perform noticeably better (up to 98%) than previously reported surfactants,^44^ which normally display *K*_D_ values between 70 and 90% under comparable testing conditions. This enhancement is attributed to the dual-head and dual-tail configuration of cationic gemini surfactants, which allows for lower critical micelle concentrations (CMC), stronger surface activity, and more efficient disruption of oil films.

### 3.4. Antimicrobial activity

The antibacterial efficacy of the four synthesized Gemini cationic surfactants (4a–d) was assessed against *L. monocytogenes*, *S. aureus*, *E. coli*, *Salmonella* sp., and *C. albicans* after 24 hours. The inhibitory zone results indicate significant variations among the tested compounds. Compound 4d demonstrated the largest inhibition zones (14–16 mm) against all assessed bacteria. Gram-positive bacteria (*Listeria monocytogenes*, *Staphylococcus aureus*) demonstrated higher sensitivity compared to Gram-negative strains (*Escherichia coli*, *Salmonella* sp.). The inhibition zones were measured at 12, 15 mm for *L. monocytogenes* and *S. aureus*, and 13, 16 mm for *E. coli* and *Salmonella* sp., respectively. Conversely, *Candida albicans* had the smallest inhibitory zones (12–14 mm).

Kowalczyk *et al.*^45^ revealed that gemini surfactants exhibit potent antibacterial properties due to their distinctive molecular architecture, facilitating the disruption of microbial cell membranes. The hydrophobic tails and dual cationic head groups enhance interactions with negatively charged bacterial surfaces, augmenting membrane permeability and inducing cell lysis. Our findings demonstrated that gemini surfactants exhibit broad-spectrum efficacy against both Gram-positive and Gram-negative bacteria and fungi, corroborating previous studies.^46,47^ Their strong surface activity and low critical micelle concentration (CMC) make them useful in disinfectants, personal care products, and biomedical applications.^48^

**Fig. 6:**
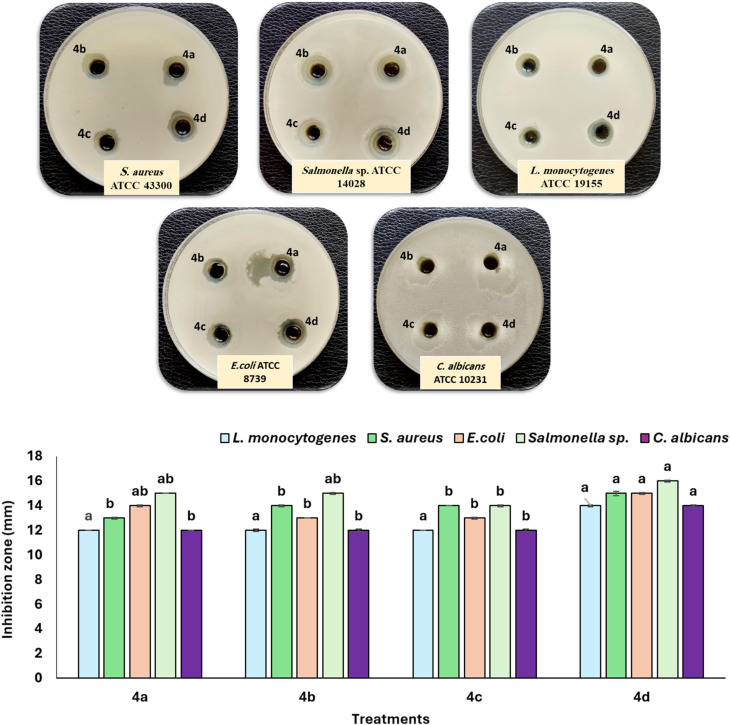
Antimicrobial activities of four ionic liquid-based Gemini surfactants (4a, 4b, 4c, and 4d) against several pathogenic microorganisms. ^*a,b^ values in the above column with the same letter do not differ significantly according to Duncan's test at the 5% level. Bar indicates ±standard deviation.

McDowell *et al.*^49^ elucidated that the molecular structure of gemini surfactants, particularly cationic 4-aminopyridinium headgroups like octenidine dihydrochloride (OCT), enables a unique integration of electrostatic, hydrogen bonding, π system, and hydrophobic interactions. These synergistic forces enhance binding affinity to lipopolysaccharides (LPS) in the outer membrane of Gram-negative bacteria, thereby increasing antimicrobial effectiveness. Similarly, Yasser *et al.*^41^ documented the synthesis of novel bioactive gemini cationic surfactants (5a–d), incorporating a benzoimidazole moiety, designed for application as petroleum dispersants and antimicrobial agents through an efficient synthetic methodology. These compounds exhibited considerable antibacterial activity, characterized by inhibition zone diameters ranging from 36 to 40 mm. Additionally, Pérez *et al.*^50^ also tested the arginine-based gemini surfactant C9(LA)2 against strains of *Candida albicans* and *Escherichia coli* that were resistant to antibiotics. The compound demonstrated substantial efficacy in eliminating resistant *C. albicans* strains and moderate effectiveness against resistant *E. coli*, underscoring its potential as a candidate for combating drug-resistant infections (Fig. 6).

## 4. Conclusion

One of the promising compounds is ionic liquid-based Gemini surfactants with a wide range of applications, such as antimicrobial agents, petro-collecting and dispersing agents. This study was based on waste recycling to obtain sustainable materials with different applications. The ionic liquid-based Gemini cationic surfactants (ILGCS) (4a–d) were obtained *via* four steps of a synthetic route. The materials obtained were characterized by different spectroscopic techniques such as FT-IR, ^1^H NMR, and ^13^C NMR. The surface activity of the synthesized surfactants (4a–d) was evaluated using their critical micelle concentration (CMC), the surface tension, and the surface pressure. Results concluded that among the four compounds, compound 4d demonstrates the highest surface activity based on its lowest value of CMC, the largest surface pressure, and the lowest surface tension at micellization. These results highlight the effectiveness of the structural design in improving surfactant performance and suggest that 4d is the most promising candidate for further applications requiring strong surface activity, such as emulsification, detergency, or solubilization processes. The petro-collecting and dispersing properties of ILGCS (4a–d) were utilized on three different water sources that differ in salinity in diluted and undiluted form. It was noted that the Gemini surfactants exhibited higher efficiency as petro-dispersing agents than as petro-collecting agents in the diluted form, with their performance influenced by the length of the spacer group. As well as all the synthesized gemini surfactants exhibited outstanding petroleum-dispersing efficiency in freshwater, with dispersion coefficient (*K*_D_) ranging from 94.5 to 93.3% for compound 4a and from 97.8 to 95.5% for compound 4d, while compounds 4b and 4c maintained stable and consistent dispersing performance for more than three days. All the newly synthesized gemini surfactants showed petroleum-dispersing activity in saltwater, although compound 4c performed better than the other surfactants. Additionally, the petroleum-collecting indices of the gemini surfactants were noted in distilled water, with *K* values ranging from 9.87 to 8.60 and 5.36. However, these collecting effects were limited to the first 24 h, after which the petro-dispersing properties became dominant and persisted for more than three days. The synthesized ionic liquid-based Gemini surfactants (4a, 4b, 4c, and 4d) exhibited notable antimicrobial activity against a range of pathogenic microorganisms after 24 h of exposure. Compound 4d, with the longest linker and spacer, consistently showed superior efficacy, particularly against Gram-positive bacteria. The results confirm that molecular flexibility and hydrophobic surface area play a critical role in enhancing membrane disruption. These results support the potential of ionic liquid-based gemini surfactants as rapid and broad-spectrum antimicrobial agents, with promising applications in medical, pharmaceutical, and industrial settings.

## Conflicts of interest

All authors declare that they have no conflict of interest.

## Supplementary Material

RA-016-D5RA09876A-s001

## Data Availability

All data are available upon request from the authors. Supplementary information (SI) is available. See DOI: https://doi.org/10.1039/d5ra09876a.
