# Supplementary material for: Eco-friendly and sustainable gemini ionic liquid surfactants derived from coffee waste: design, surface activity, oil spill treatment, and antimicrobial performance
Source: RSC Adv. 2026 Jan 14;16(4):3231–43. doi: 10.1039/d5ra09876a (PMC12801401; doi:10.1039/d5ra09876a)
Supplement: RA-016-D5RA09876A-s001 [file RA-016-D5RA09876A-s001.pdf]

Supplementary Information (SI) for RSC Advances.
This journal is © The Royal Society of Chemistry 2026

Supporting information

Figure S1. ^{1}H and ^{13}C NMR spectra *N,N'-((ethane-1,2-diylbis(oxy))bis(2-oxoethane-2,1-diyl))bis(N,N-dimethyl-3-tetradecanamidopropan-1-aminium) chloride (**4a**)*.

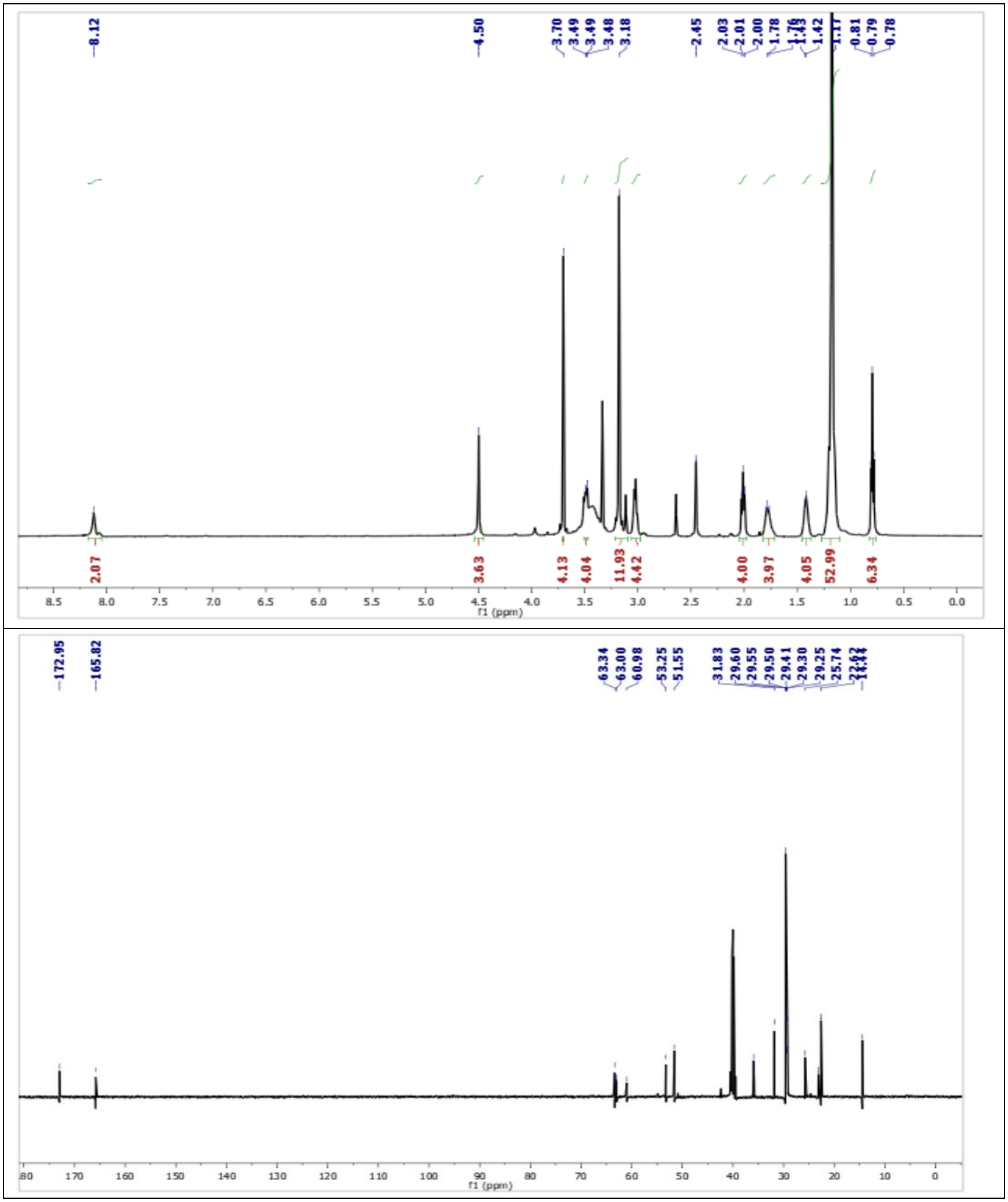

Figure S2. ^{1}H and ^{13}C NMR spectra *N,N'-((propane-1,3-diylbis(oxy))bis(2-oxoethane-2,1-diyl))bis(N,N-dimethyl-3-tetradecanamidopropan-1-aminium) chloride (**4b**).*

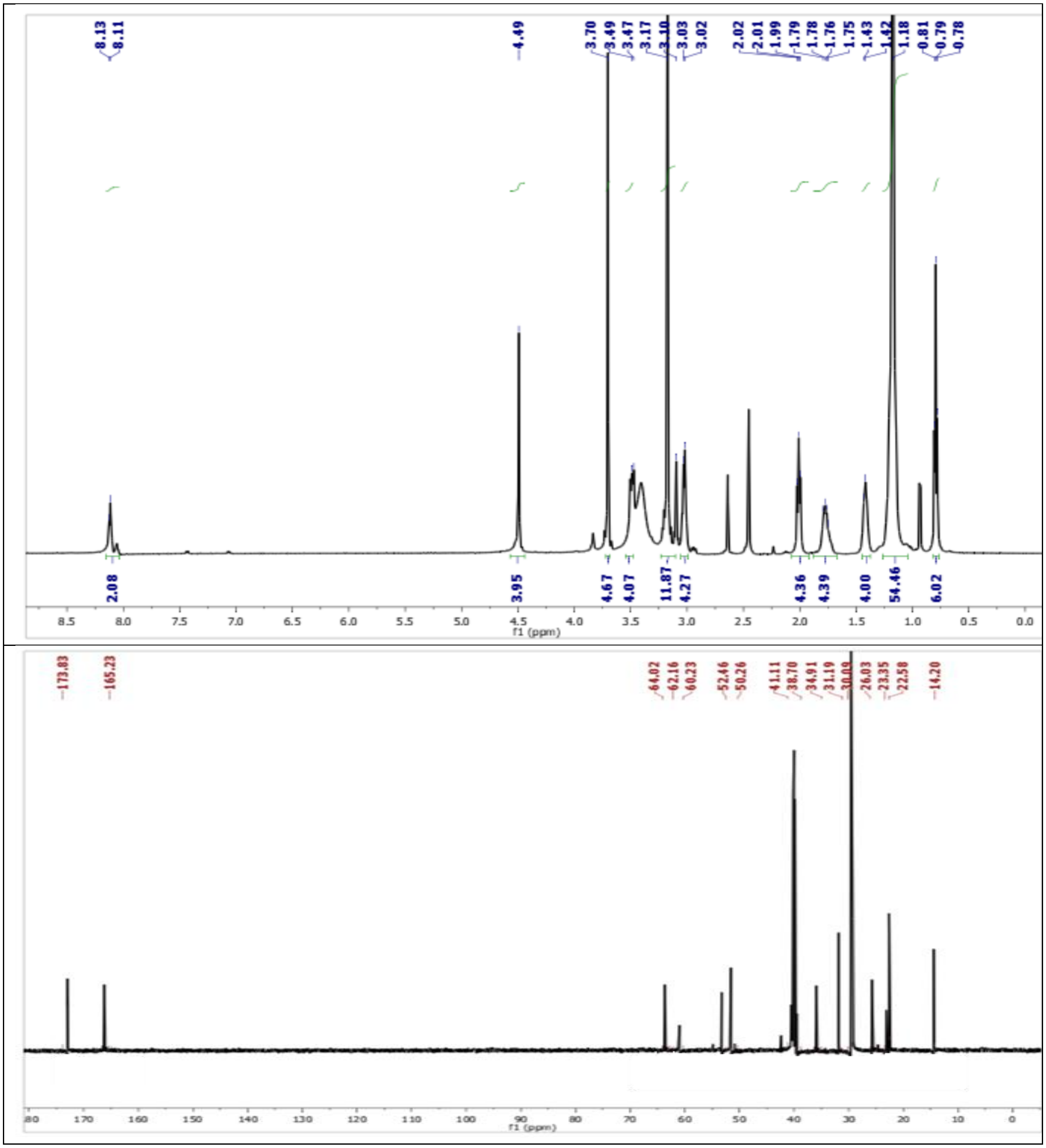

Figure S3. ^{1}H and ^{13}C NMR spectra of *N,N'-((pentane-1,5-diylbis(oxy))bis(2-oxoethane-2,1-diyl))bis(N,N-dimethyl-3-tetradecanamidopropan-1-aminium) chloride (**4c**)*.

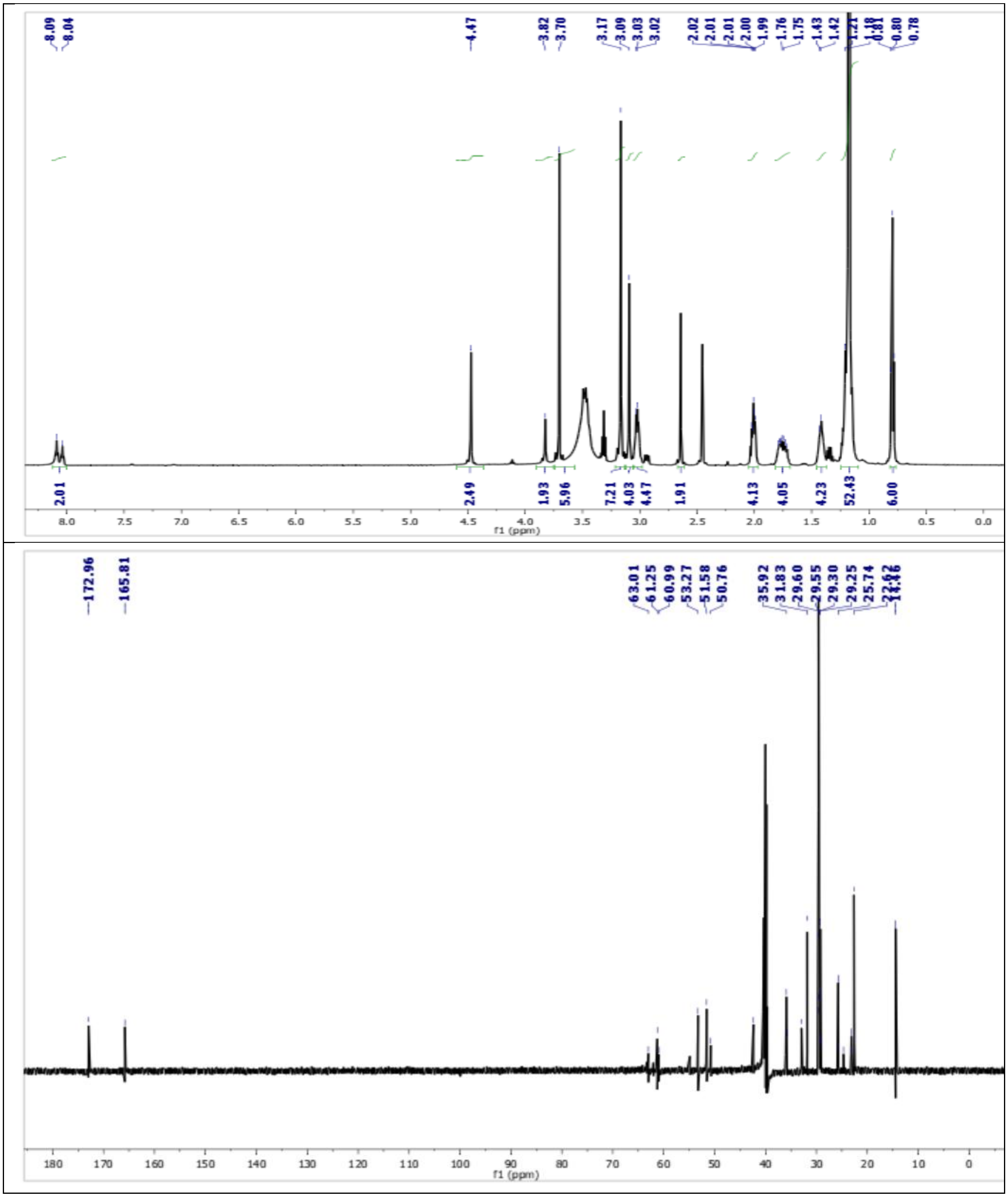

Figure S4. ^{1}H and ^{13}C NMR spectra of *N,N'-((hexane-1,6-diylbis(oxy))bis(2-oxoethane-2,1-diyl))bis(N,N-dimethyl-3-tetradecanamidopropan-1-aminium) chloride (**4d**)*.

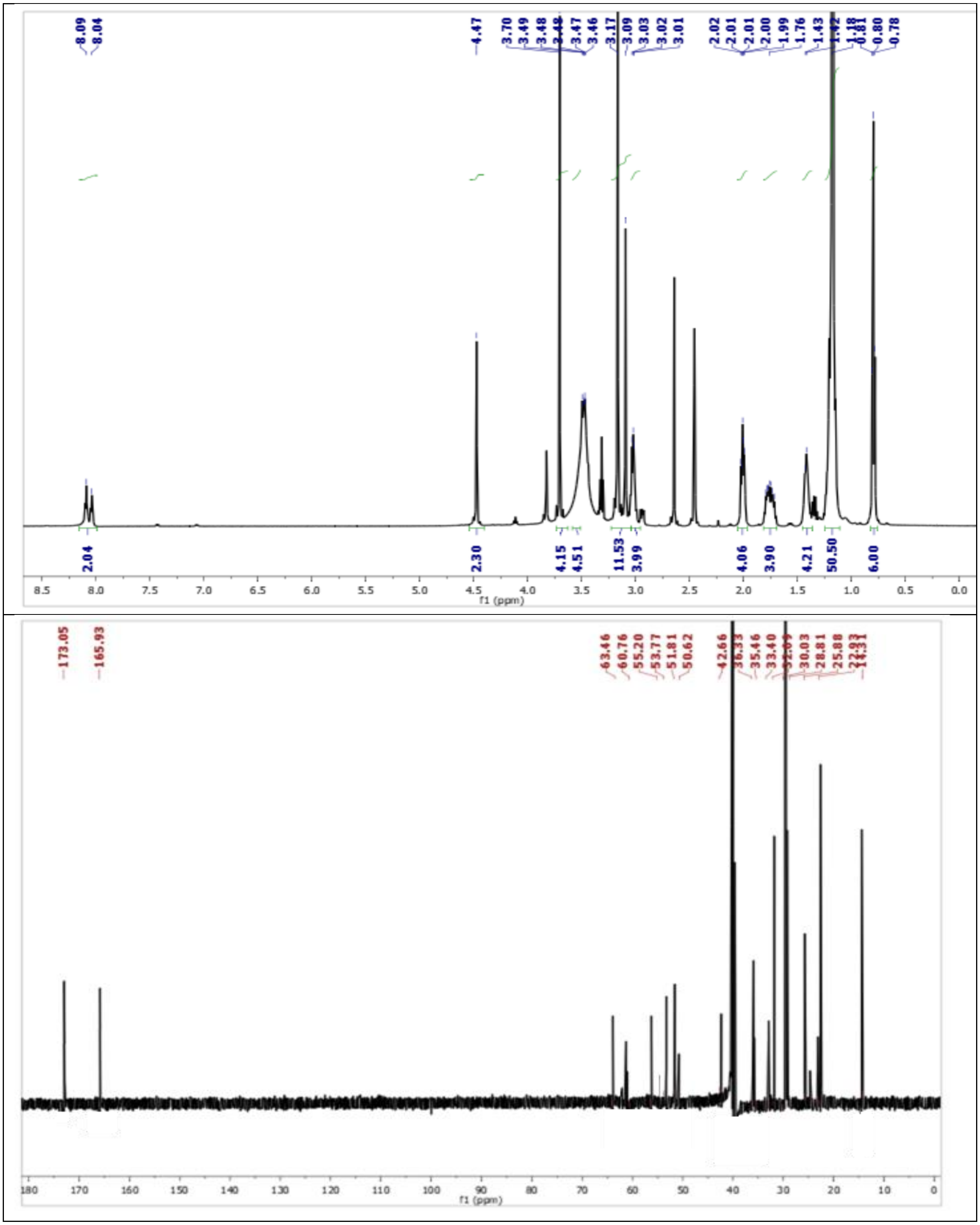

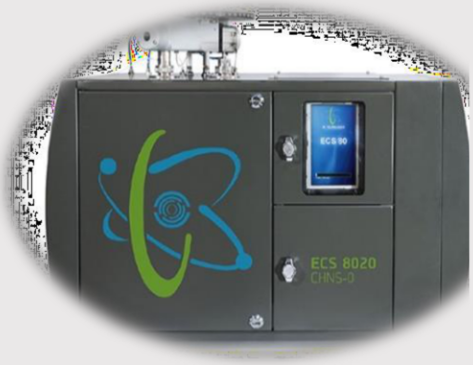

PROFILENC TECHNOLOGIES ECS 8020 CHNS-O ELEMENTAL COMBUSTION SYSTEM – ELEMENTAL ANALYZER

The ECS 8020 is a C, H, N, S, O Elemental Analyzer Model based on the Dumas combustion method. It represents an evolution of elemental analysis techniques based on sample combustion and chromatographic separation. The combustion product, i.e. CO_2, H_2O, SO_2 and N_2 are separated in a GC column and quantified by a high resolution TCD Detector. ECS 8020 offers the possibility to set several configurations for the determination of the target element; possible configurations are CHNS, CHN, CN, CNS, and O. The adaptability of ECS 8020 could also be expressed in terms of sample type and size. A wide type of samples, both liquids and solids, in wide sizes, from micrograms to grams of organic materials, can be analyzed. The ECS 8020 is particularly suitable for linking to other units for determination of the isotopic ratios of stable isotopes in elements.

Sample	C (wt%)	H (wt%)	N (wt%)	O (wt%)	Cl (wt%)
**4a**	63.9 ± 0.3	10.1 ± 0.2	5.9 ± 0.2	10.1	7.4 ± 0.5
**4b**	64.6 ± 0.3	10.2 ± 0.2	5.7 ± 0.2	9.8	7.2 ± 0.5
**4c**	65.1 ± 0.3	10.5 ± 0.2	5.3 ± 0.2	9.2	6.9 ± 0.5
**4d**	65.3 ± 0.3	10.8 ± 0.2	5.1 ± 0.2	8.9	6.8 ± 0.5
